# TLC-bioautography-guided valorization of vetiver (*Chrysopogon* spp.) leaf extracts into an anti-acne gel

**DOI:** 10.1186/s40643-025-00941-1

**Published:** 2025-09-17

**Authors:** Duangsamorn Boonwun Boyd, Lapatrada Mungmai, Sornkanok Vimolmangkang, Sirada Uputinan, Dutrudi Panprommin, Wasu Pathom-aree, Panitnart Auputinan

**Affiliations:** 1 Department of Biological Sciences, Louisiana State University, Baton Rouge, LA 70803 USA; 2 Department of Cosmetic Sciences, School of Pharmaceutical Sciences, University of Phayao, Phayao, 56000 Thailand; 3 Department of Pharmacognosy and Pharmaceutical Botany, Faculty of Pharmaceutical Sciences, Chulalongkorn University, Bangkok, 10330 Thailand; 4 PhytoAnalytica Testing Laboratory, Leapdelab Company Limited, Samut Prakan, 10130 Thailand; 5 Division of Otolaryngology, Sukhothai Hospital, Sukhothai, 64000 Thailand; 6 Division of Fisheries, School of Agriculture and Natural Resources, University of Phayao, Phayao, 56000 Thailand; 7 Unit of Excellence Biotechnology for Sustainable Agricultural Development (Fundamental Fund 2025, Grant No. 5051/2567), University of Phayao, Phayao, 56000 Thailand; 8 Department of Biology, Faculty of Science, Chiang Mai University, Chiang Mai, 50200 Thailand; 9 Department of Biotechnology, School of Agriculture and Natural Resources, University of Phayao, Phayao, 56000 Thailand

**Keywords:** *Chrysopogon* spp, Leaf biomass, Antimicrobial resistance, TLC-bioautography, Bioactivity-guided fractionation, Anti-acne gel, Plant-based formulation, Phytochemical valorization

## Abstract

The growing issue of antimicrobial resistance (AMR) in acne-related bacteria continues to undermine the effectiveness of standard treatments. This study examined the antibacterial potential of *Chrysopogon* spp. leaf biomass. The leaves, which are often discarded or used locally as mulch to help retain soil moisture, are an underused agricultural byproduct. Leaf extracts were obtained from five cultivars using different ethanol and water-based solvent systems and were screened against *Cutibacterium acnes*,* Staphylococcus aureus*, and *Streptococcus pyogenes*. The 50% ethanolic extracts showed the strongest and most consistent inhibition patterns, with MIC values ranging from 7.81 to 125 mg/mL. TLC-bioautography was employed to identify active components, which were primarily located within the mid-polar Rf range. Topical gels were developed using 4% and 5% w/w of the most active extract showing the strongest antibacterial effect, which was found in the Huai Kha Khang cultivar. The 5% gel exhibited inhibition zones of 6–20 mm, stayed stable and maintained its antimicrobial activity after four weeks of storage at 4 ± 1 °C, 25 ± 2 °C, and 45 ± 2 °C. These findings highlight the therapeutic potential of underutilized vetiver leaves as a plant-based resource for anti-acne gel formulations in AMR-sensitive applications.

**Graphical Abstract:**

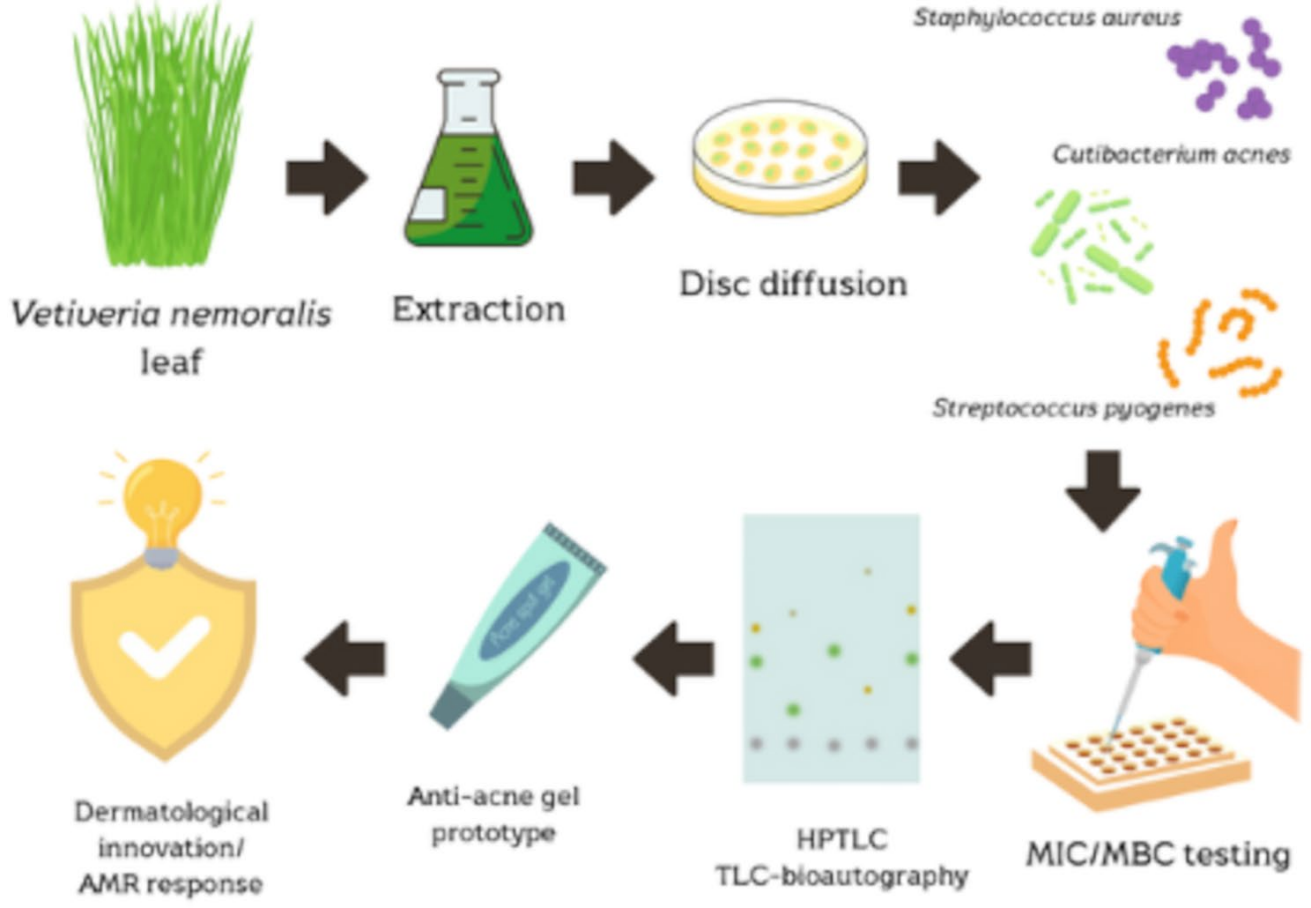

## Introduction

Acne vulgaris is a prevalent inflammatory skin condition that usually develops during adolescence and continues into early adulthood. Treatment often begins with topical applications, while oral or systemic agents are added when symptoms become more persistent. Standard acne treatment often involves the use of antimicrobials, synthetic agents, and anti-inflammatory drugs. Current practices have shifted to combining multiple agents, such as using two antibiotics or pairing antimicrobial drugs with synthetic adjuncts (Abozeid et al. 2023).

Treating acne is becoming more difficult as resistance to antibiotics emerges among several common skin bacteria (Dréno et al. 2018, 2020; Schafer et al. 2013; Walsh et al. 2016). The rise of antibiotic-resistant acne bacteria reflects a pattern that has also been noted in other areas of AMR (World Health Organization 2020). Several acne-associated bacteria, including *Cutibacterium acnes*,* Staphylococcus aureus*,* and Streptococcus pyogenes* have shown reduced responsiveness to treatments that were previously effective. As part of ongoing attempts to find alternatives, recent studies have renewed interest in plant-based compounds. A number of these natural materials have shown antibacterial potential and could be relevant to future dermatological applications.

In light of these challenges, natural agents are now being studied in dermatology as part of broader strategies to shift away from conventional antibiotic reliance. Natural antibacterial compounds from plants are being explored with increasing scientific interest because they exhibit multiple mechanisms of action and are less likely to promote resistance. A number of medicinal plants carry distinct chemical profiles that are regarded as promising reservoirs of antibacterial agents (Chomnawang et al. 2005; Ebob et al. 2021; Newman and Cragg 2020). Numerous agro-industrial as well as agricultural by-products possess the capacity for further processing into products of greater commercial importance (Srianta et al. 2021). Sustainable material innovation now requires converting agricultural waste into bioactive products to implement resource management strategies through circular systems (Saini et al. 2019). One example is *Chrysopogon* spp., a tropical grass widely cultivated for its aromatic roots, which have long been valued in traditional preparations for their calming and antimicrobial properties (Luqman 2012). Harvesting the roots may reduce the plant’s contribution to erosion control, especially on sloped terrain (Chomchalow 2001). However, the leaves are often discarded or repurposed as local mulch. Some early findings suggest that vetiver leaf extracts may inhibit bacterial growth, but their potential in topical dermatological formulations remains largely unexplored (Rathnayake et al. 2020). In particular, their possible use in acne treatment has not been examined in detail. This overlooked material could offer a way to develop functional bioproducts from plant waste that is otherwise unused. In this study, the leaves were chosen instead of the roots because they are more sustainably harvested and are typically treated as agricultural waste. This approach supports the valorization of underused plant materials and reflects a growing interest in circular bioeconomy practices. Five distinct cultivars were included to capture phytochemical variation and enable comparative evaluation of their antibacterial potential.

TLC-bioautography has previously been applied in studies examining bioactive constituents from vetiver roots. For example, Soni and Dahiya (2015) assessed general microbial effects, without extending the work to acne-relevant applications. In contrast, research on leaf-based extracts remains uncommon, and their contribution to topical product development remains largely unexamined.

In order to close this research gap, the present work evaluated the antibacterial activity of vetiver leaf extracts against acne-associated bacteria and localized the bioactive substances using TLC-bioautography. Extracts exhibiting the strongest antibacterial activity were advanced through a streamlined and stepwise process, beginning with crude extract preparation, followed by gel formulation, and concluding with evaluation of physical stability and antimicrobial performance. This sustainable approach highlights the potential of converting underutilized plant biomass into dermatological products, especially in therapeutic contexts affected by AMR.

To demonstrate this potential through a systematic process, the present study screened five vetiver cultivars for antibacterial activity (via disc diffusion and Minimal Inhibitory Concentration (MIC) and Minimal Bactericidal Concentration (MBC)), followed by chromatographic profiling (HPTLC), and bioactivity localization using TLC-bioautography. The most active extract was incorporated into a gel prototype, which underwent four-week stability testing and was re-evaluated for antibacterial efficacy post-formulation. This bioactivity-guided pipeline enabled a rational transition from crude extract to functional topical gel.

## Materials and methods

### Plant material and extraction

Fresh leaves from five cultivars belonging to two species, *Chrysopogon zizanioides* (L.) Roberty (Mae Hong Son (MHS), Sri Lanka (SL), Phraratchathan (PT), Maetia (MT)) and *Chrysopogon nemoralis* (Balansa) Holttum (Huai Kha Khaeng (HKK)), were collected from the Huai Hongkhrai Royal Development Study Centre located in Chiang Mai, Thailand. After visible debris was removed, the leaves were washed, naturally shade-dried, followed by oven-drying at 55 °C until fully dried and subsequently ground. Extraction was carried out with distilled water and ethanol at concentrations of 50%, 70% and 95%. The powdered leaves were extracted with each solvent at a 1:5 (w/v) ratio using an incubator shaker at 30 °C and 120 rpm for 24 h to promote active compound diffusion. After extraction, the mixture was filtered through Whatman No.1 filter paper to separate the leaf residues. The extraction process was repeated twice under the same conditions, resulting in a total of three successive extracts per sample. All filtrates were combined and concentrated using a rotary evaporator under reduced pressure and then freeze-dried. The dried extracts were reconstituted in dimethyl sulfoxide (DMSO) to a final concentration of 500 mg/mL. All samples were kept in sterile, screw-capped bottles at 4 °C until used in analytical procedures (Ganjewala and Gupta 2013). These pretreatment and extraction conditions were selected to preserve bioactive integrity and ensure representative chemical recovery across polarity classes. Shade-drying followed by controlled oven-drying was used to minimize phytochemical degradation. The ethanol gradient enabled targeted extraction of antibacterial compounds with different solubility characteristics. Maceration over 72 h ensured adequate exposure time for compound diffusion and yield optimization. This polarity-guided extraction strategy supports subsequent screening and bioautographic profiling steps. The percentage yield was calculated using the following equation:

$${\rm{Yield}}\:\left( {\rm{\% }} \right)\: = \:\frac{{{\rm{Weight}}\:{\rm{of}}\:{\rm{dry}}\:{\rm{extract}}}}{{{\rm{Weight}}\:{\rm{of}}\:{\rm{dry}}\:{\rm{plant}}}}\:{\rm{x}}\:{\rm{100}}$$

### Bacterial strains and culture conditions

Three bacterial strains were used in this study: *Cutibacterium acnes* DMST 14916, *Staphylococcus aureus* DMST 8840, and *Streptococcus pyogenes* DMST 30653. All strains were sourced from the Department of Medical Science, Ministry of Public Health, Thailand. Before testing, each strain was cultivated under specific growth conditions. Anaerobic cultivation of *C. acnes* was performed on Fluid Thioglycolate agar (FTA) at 37 °C for 72 h. In contrast, *S. aureus* was cultured aerobically in Tryptic Soy Broth (TSB) at 37 °C for 18 to 24 h. *S. pyogenes* was cultured on Brain Heart Infusion (BHI) agar, then incubated under 5% CO₂ at 37 °C for 24 h.

### Antibacterial activity assay

Colonies of each bacterial strain were suspended until the turbidity was visually adjusted to approximate a 0.5 McFarland standard. *S. aureus* and *S. pyogenes* were inoculated onto Mueller-Hinton Agar (MHA), while *C. acnes* was swabbed onto FTA for agar preparation. Sterile 6 mm paper discs were saturated with 30 µL of each extract (500 mg/mL) and placed on the prepared plates. Incubation was carried out at 37 °C for 24–72 h, depending on the specific bacterial strain. DMSO served as the negative control, while clindamycin (2 µg, Oxoid™, UK) was used as the positive control. The diameter of inhibition zones was measured after incubation. MIC and MBC values were determined using the broth microdilution method in accordance with CLSI guidelines (Clinical and Laboratory Standards Institute 2016).

### High-performance thin layer chromatography (HPTLC)

To generate a phytochemical profile of vetiver leaf extracts, HPTLC was used as the analytical technique. Selection of samples was guided by previous antibacterial screening, which highlighted the activity of 50% ethanolic extracts from five cultivars. Before application, each extract was diluted in its corresponding solvent to achieve a final concentration of 10 mg/mL.

Each sample (5 µL) was applied as a 6 mm band onto silica gel 60 F254 HPTLC plates (20 × 10 cm, Merck, Germany) using a Linomat 5 applicator (CAMAG, Switzerland). Bands were positioned 8 mm from the bottom edge of the plate (baseline) and spaced 10 mm apart to prevent overlapping. Several solvent mixtures were evaluated to identify the mobile phase offering optimal separation of phytochemical constituents. The systems tested included dichloromethane:methanol (93:7), toluene:ethyl acetate (93:7), toluene:acetonitrile:ethyl acetate (9:5:2), and ethyl acetate:water:formic acid:acetic acid (100:21:11:11). Chromatographic development was performed in a CAMAG ADC-2 automatic development chamber pre-saturated with the mobile phase vapor for 20 min at 33% relative humidity. The migration distance was set to 70 mm. After development, the plates were dried using a gentle air stream for 5 min before visualization.

After drying, the plates were inspected under UV light at 254 and 366 nm. For post-chromatographic derivatization, anisaldehyde–sulfuric acid reagent was freshly prepared by mixing 0.5 mL p-anisaldehyde, 10 mL glacial acetic acid, 85 mL methanol, and 5 mL concentrated sulfuric acid, following the protocol of Reich and Schibli (2007). Plates were sprayed evenly and heated at 105 °C for 5 min to induce chromophore development. For the ethyl acetate:water:formic acid:acetic acid mobile phase, natural product/polyethylene glycol (NP/PEG) reagent was used instead. Derivatization involved sequential spraying with 1% w/v 2-aminoethyl diphenylborinate in methanol (NP reagent) and 5% w/v polyethylene glycol 400 in ethanol (PEG reagent), as described by Wagner and Bladt (1996), followed by heating under the same conditions. After derivatization, chromatographic bands were visualized using the CAMAG TLC visualizer under visible light as well as UV light at 254 and 366 nm. Observations of band clarity, intensity, and chromogenic variation were digitally recorded and used to compare phytochemical expression across the five cultivars.

### TLC-bioautography

TLC-bioautography was conducted to visualize antibacterial constituents contained in selected vetiver extracts. Based on prior antimicrobial screening, the 50% ethanolic extracts from Huai Kha Khaeng (HKK) and Mae Hong Son (MHS) cultivars exhibited notable inhibitory effects and were subsequently examined using a modified agar-overlay method (Jesionek et al. 2017).

For sample preparation, each crude extract was reconstituted in 50% ethanol to produce a concentration series (10, 20, 30, 40, 50, 60, and 70 mg/mL). Aliquots (*5* µL) of each concentration were spotted onto silica gel TLC plates and developed using the optimized mobile phase of toluene:acetonitrile:ethyl acetate (9:5:2), as established in prior HPTLC analysis. Clindamycin (2 µL, 0.01 mg/mL) served as the positive control.

After chromatographic development and air-drying, the plates were immersed for 10 seconds in bacterial suspensions adjusted to a 0.5 McFarland standard and incubated at 37*°*C for 17 hours. Zones of inhibition were visualized by spraying 2,3,5-triphenyltetrazolium chloride (TTC) evenly across the plate, followed by incubation in the dark for 2 to 4 h to increase contrast and enhance zone visibility.

The inhibition zones were aligned with corresponding chromatographic positions under visible and UV light (254 and 366 nm). Retention factor (Rf) values, calculated as the ratio of the distance travelled by the compound to that of the solvent front (Rf = distance of compound / distance of solvent front), were used to link antibacterial activity with phytochemical locations. Differences in band clarity, color tone, and migration across solvent systems facilitated the identification of bioactive regions, which guided the selection of promising regions for incorporation into the final gel prototypes.

This method is adapted from established bioautographic procedures and is not novel per se. The underlying principle involves diffusion of bioactive compounds from the developed chromatogram into an overlaid agar inoculated with bacteria. Following incubation, 1% TTC is applied as a redox indicator that turns purple or red in areas of active bacterial metabolism. Therefore, inhibition zones appear as colorless or pale regions on a purple background, reflecting localized suppression of bacterial growth due to active compounds.

### Gel formulation and stability testing

#### Development of synthetic gel base for extract incorporation

A synthetic gel base was systematically developed for the incorporation of active extracts using a mild, solvent-free approach. The formulation included ammonium acryloyldimethyltaurate, diazolidinyl urea, iodopropynyl butylcarbamate, propylene glycol, and purified water (Table 1). Initially, diazolidinyl urea and iodopropynyl butylcarbamate were mixed, followed by the addition of propylene glycol and purified water. The mixture was stirred until fully homogeneous, after which ammonium acryloyldimethyltaurate was added gradually under continuous magnetic agitation to initiate gelation. The resulting gel was then left to equilibrate at ambient temperature for 24 h, allowing complete hydration and the development of a uniform and stable matrix.

The gel base was stored for four weeks at 4 ± 1 °C, 25 ± 2 °C, and 45 ± 2 °C to assess physical integrity. Its texture, clarity, pH, and viscosity were observed throughout the period (Dantas et al. 2016). Results from these observations indicated that the formulation maintained stability and remained suitable for later incorporation of active extracts. This final formulation provided a consistent, hydrated gel network capable of accommodating crude extracts without structural disruption.

**Table 1:** Gel base formulation

Formulas	Ammonium acryloyldimethyltaurate (%w/w)	Diazolidinyl Urea (and) Iodopropynyl Butylcarbamate (and) Propylene Glycol (%w/w)	Water (%w/w)
A	1.7	0.5	To 100
B	2.0	0.5	To 100
C	2.5	0.5	To 100

### Extract incorporation and formulation evaluation

The optimization process of gel base development led to the selection of Formulation B, which demonstrated superior clarity and desirable rheological behavior. Formulation B was selected as the final gel base owing to its optimal balance between viscosity and spreadability, as well as its compatibility with vetiver leaf extract—preserving antibacterial function while maintaining physicochemical stability. The concentration of ammonium acryloyldimethyltaurate in this formulation yielded a stable, hydrated gel matrix suitable for dermal application, without compromising texture or structural integrity. Based on antimicrobial activity results, the Huai Kha Khaeng (HKK) crude extract exhibited stronger and more consistent antibacterial effects across all tested strains compared to MHS and was therefore chosen for incorporation (Table 2). To ensure homogeneous dispersion, the HKK crude extract was pre-dissolved in propylene glycol and gradually incorporated into Formulation B under continuous stirring, allowing the formulation to reach a uniform and stable consistency.

A four-week stability test was performed on the extract-containing gel under three storage conditions: 4 ± 1 °C, 25 ± 2 °C, and 45 ± 2 °C. Stability investigations focused on key characteristics such as pH, viscosity, color, phase separation, and antibacterial activity, all assessed at weekly intervals (Gunes et al. 2017). Antimicrobial testing included direct comparisons between the vetiver gel formulations (B1 and B2) and standard treatments, namely a commercial clindamycin phosphate gel (1% w/w), a clindamycin disc (2 µg, Oxoid™, UK), and propylene glycol as the negative control.

This setup allowed observation of both therapeutic response and formulation integrity across all samples. Overall, this process provides a practical way to convert plant-based resources into antibacterial gels through a direct, bioactivity-guided formulation route.

### Statistical analysis

All experiments were performed in triplicate. Results are expressed as mean ± SD and analyzed using one-way analysis of variance (ANOVA) with SPSS software (Version 22.0; IBM, New York, U.S.). Differences among sample means were determined using Duncan’s multiple range test, with statistical significance set at *p* < 0.05. For each table, different superscript letters within the same column indicate significant differences among samples.

**Table 2:** Vetiver extract gel formulation

Formulas	HKK crude extract (%w/w)	Ammonium acryloyldimethyltaurate (%w/w)	Diazolidinyl Urea (and) Iodopropynyl Butylcarbamate (and) Propylene Glycol (%w/w)	Water (%w/w)
B1	4.0	2.0	0.5	To 100
B2	5.0	2.0	0.5	To 100

## Results

### Extraction yield of vetiver leaf extracts

The extraction yields of crude vetiver leaf extracts obtained using various solvents from five cultivars are shown in Table 3. Among the solvent systems tested, 50% ethanol consistently produced the highest yields across most cultivars, surpassing those of 70% ethanol, distilled water, and 95% ethanol. The SL cultivar gave the maximum yield at 12.40% when extracted with 50% ethanol, while the MHS cultivar yielded the lowest at 1.47% under 95% ethanol extraction. These findings suggest that 50% ethanol is a suitable solvent concentration for extracting diverse phytoconstituents from vetiver leaves since it proficiently solubilizes both polar and lipophilic compounds. This polarity-dependent solubility profile guided subsequent solvent selection for downstream bioassay testing.

**Table 3:** Percentage yield of vetiver leaf extracts (%) obtained using different solvents

Cultivar	Water	50% EtOH	70% EtOH	95% EtOH
PT	5.00	7.95	6.40	2.88
MT	5.65	10.25	8.28	4.29
HKK	6.98	8.30	7.40	2.43
MHS	6.54	8.26	6.71	1.47
SL	7.26	12.40	10.04	4.35

Note: EtOH = Ethanol

### Antibacterial activity of vetiver leaf extracts

**Fig. 1:**
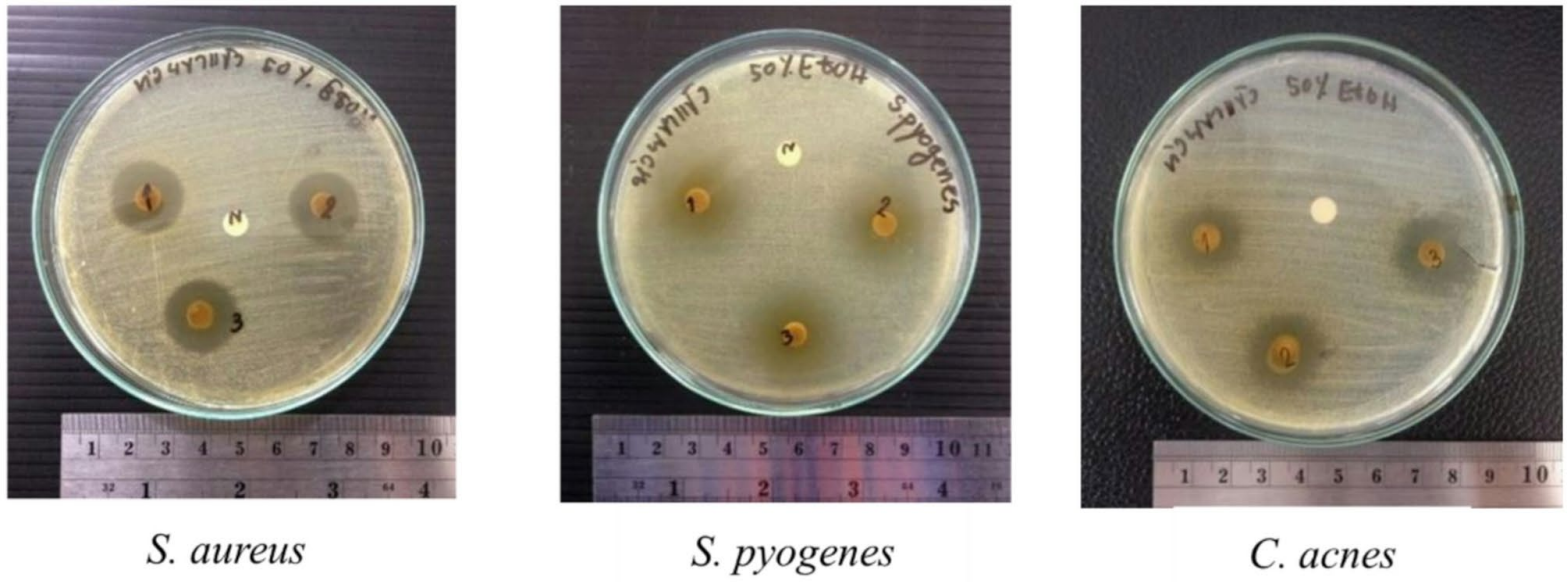
Representative images of disc diffusion assay showing inhibition zones produced by 50% ethanolic extract of *Chrysopogon nemoralis* (HKK cultivar) against *Staphylococcus aureus*,* Streptococcus pyogenes*, and *Cutibacterium acnes*. Crude extracts were tested in triplicate on MHA or FTA for *C. acnes*, with inhibition zones visible around each disc. The center disc (N) served as negative control (DMSO only)

Antibacterial screening of vetiver leaf extracts was carried out using the disc diffusion method against *S. pyogenes*, *S. aureus*, and *C. acnes* (Table 4). Among the samples, the 50% ethanolic extract from HKK produced the largest and most reproducible zones of inhibition, measuring 17.67 ± 0.29 mm against *S. aureus* and 15.00 ± 1.00 mm against *C. acnes* (Fig. 1.). The MHS extract from 95% ethanol also inhibited *S. pyogenes* effectively, with an average zone of 17.33 ± 2.52 mm (*p* < 0.05).

In contrast, water-based extracts showed no observable antibacterial activity. Clindamycin, as the positive control, yielded broader inhibition zones across all test bacteria. Moderate inhibition was also observed with the 70% ethanolic extract of MHS against *C. acnes* (11.33 ± 1.04 mm), though other cultivars showed variable activity. Altogether, the consistent antibacterial effect observed in the HKK extract supports its suitability for further phytochemical analysis and development into a topical gel formulation.

### Minimum inhibitory and bactericidal concentrations (MIC and MBC)

The minimum inhibitory concentrations (MICs) and minimum bactericidal concentrations (MBCs) of crude vetiver leaf extracts against *S. pyogenes*,* S. aureus*, and *C. acnes* are summarized in Table 5. The 50% ethanolic extract of the SL cultivar exhibited the strongest antibacterial activity against *C. acnes*, with both MIC and MBC values determined at 7.81 mg/mL. The HKK extract showed low MIC values of 7.81 mg/mL against both *S. pyogenes* and *C. acnes*, similar to the SL extract for *S. pyogenes*.

Water-based extracts were excluded from MIC/MBC testing because no antibacterial inhibition was observed in earlier assays. Antibacterial efficacy was enhanced when phytochemicals were extracted using 50% ethanol, likely due to its polarity balance, which facilitates the solubilization of both hydrophilic and lipophilic compounds. Bactericidal effects were frequently detected at one or two dilutions beyond the MIC values. The low MIC and MBC values of SL and HKK extracts aligned with their inhibition zones in disc diffusion assays, reinforcing their antibacterial potential. Given the favorable antimicrobial activity of the 50% ethanolic extracts across all cultivars, this solvent system was selected for subsequent phytochemical characterization using HPTLC. This solvent choice was further supported by its polarity balance, which likely facilitated the simultaneous extraction of hydrophilic and lipophilic antibacterial constituents. The high yields, coupled with low MIC/MBC values observed in multiple cultivars, indicate that 50% ethanol offered both chemical coverage and biological potency. These combined efficiencies justify its use in downstream fingerprinting and formulation steps.

**Table 4:** Diameter of Inhibition zones (mm) of vetiver leaf extracts against *S. pyogenes*, *S. aureus*, and *C. acnes*

Cultivar	Solvent	*S. pyogenes*	*S. aureus*	*C. acnes*
MT	95% EtOH	11.17 ± 2.02^fgh^	12.57 ± 0.40^b^	7.67 ± 0.29^fg^
	70% EtOH	13.33 ± 0.58^def^	7.00 ± 0.50^ef^	10.50 ± 0.50^cd^
	50% EtOH	11.50 ± 1.80^fgh^	7.50 ± 1.00^e^	7.17 ± 0.58^g^
	Water	0.00 ± 0.00^i^	0.00 ± 0.00^g^	0.00 ± 0.00^h^
MHS	95% EtOH	17.33 ± 2.52^a^	8.83 ± 1.89^cd^	8.50 ± 1.00^efg^
	70% EtOH	9.50 ± 1.50^h^	7.50 ± 0.50^e^	11.33 ± 1.04^c^
	50% EtOH	12.00 ± 2.18^efg^	9.83 ± 1.04^c^	13.67 ± 0.58^b^
	Water	0.00 ± 0.00^i^	0.00 ± 0.00^g^	0.00 ± 0.00^h^
PT	95% EtOH	14.17 ± 0.58^de^	11.60 ± 0.60^b^	8.67 ± 2.02^defg^
	70% EtOH	14.83 ± 0.29^bcd^	11.83 ± 0.58^b^	7.83 ± 0.76^fg^
	50% EtOH	15.17 ± 2.47^abcd^	7.83 ± 0.58^de^	10.00 ± 1.32^cdf^
	Water	0.00 ± 0.00^k^	0.00 ± 0.00^i^	0.00 ± 0.00^h^
SL	95% EtOH	13.17 ± 1.26^fgh^	9.00 ± 0.00^ef^	9.17 ± 0.76^def^
	70% EtOH	10.50 ± 1.80^ij^	7.33 ± 0.29^g^	9.00 ± 0.87^defg^
	50% EtOH	14.33 ± 0.58^efg^	6.17 ± 0.29^h^	11.84 ± 0.29^c^
	Water	0.00 ± 0.00^k^	0.00 ± 0.00^i^	0.00 ± 0.00^h^
HKK	95% EtOH	16.67 ± 0.76^cde^	8.17 ± 0.29^fg^	10.50 ± 2.50^cd^
	70% EtOH	15.50 ± 1.50^cdef^	8.00 ± 1.00^fg^	11.33 ± 1.53^c^
	50% EtOH	16.90 ± 0.53^cd^	17.67 ± 0.29^a^	15.00 ± 1.00^b^
	Water	0.00 ± 0.00^k^	0.00 ± 0.00^i^	0.00 ± 0.00^h^
Clindamycin	-	77	27.5	88

Values are presented as mean ± SD (*n* = 3). Different superscript letters within each column indicate significant differences at *p* < 0.05 based on Tukey’s HSD test

**Table 5:** MIC and MBC values (mg/mL) of vetiver leaf extracts against *S. pyogenes*, *S. aureus*, and *C. acnes*

Cultivar	Solvent	MIC			MBC		
		*S. pyogenes*	*S. aureus*	*C. acnes*	*S. pyogenes*	*S. aureus*	*C. acnes*
MT	95% EtOH	31.625	125	62.5	62.5	125	62.5
	70% EtOH	15.625	125	125	62.5	125	125
	50% EtOH	7.81	62.5	31.25	31.25	125	31.25
MHS	95% EtOH	62.5	62.5	31.25	62.5	125	62.5
	70% EtOH	15.62	31.25	31.25	31.25	500	31.25
	50% EtOH	15.62	62.5	15.62	15.62	62.5	31.25
PT	95% EtOH	15.62	125	31.25	62.5	125	31.25
	70% EtOH	62.5	125	62.5	62.5	125	62.5
	50% EtOH	31.25	62.5	31.25	31.25	125	125
SL	95% EtOH	7.81	62.5	15.62	7.81	125	250
	70% EtOH	7.81	62.5	31.25	7.81	125	31.25
	50% EtOH	7.81	31.25	7.81	7.81	62.5	7.81
HKK	95% EtOH	31.25	31.25	7.81	125	125	7.81
	70% EtOH	7.81	62.5	31.25	15.62	125	31.25
	50% EtOH	7.81	62.5	15.62	7.81	62.5	125

Note: MIC = Minimum Inhibitory Concentration; MBC = Minimum Bactericidal Concentration. Values reported for water extracts are excluded due to absence of detectable activity in diffusion assay. Data represents the average of triplicates.

### Phytochemical separation and solvent selection via HPTLC

HPTLC analysis was conducted using four different solvent systems to evaluate which mixture provided effective separation of bioactive compounds in vetiver leaf extracts. Testing focused on 50% ethanolic extracts obtained from five cultivars with notable antibacterial performance and extraction yield. A system was considered effective when it produced distinct bands representing both non-polar and mid-polar constituents on the developed plate.

**Fig. 2:**
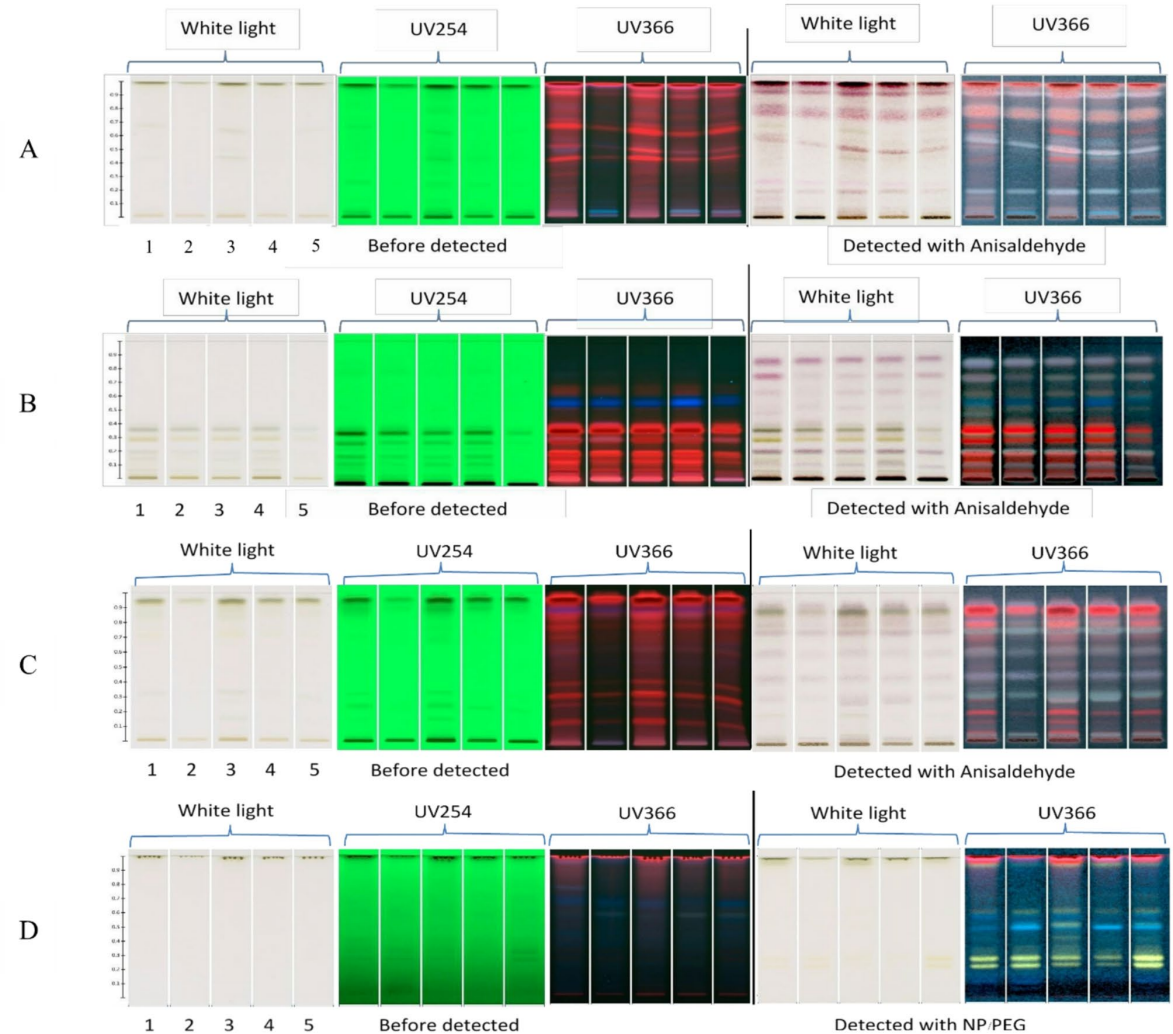
Chromatographic profiles of 50% ethanolic crude extracts from five vetiver cultivars (1: PT; 2: MT; 3: HKK; 4: MHS; 5: SL) developed using four mobile phase systems: (**A**) dichloromethane: methanol (93:7), (**B**) toluene: ethyl acetate (93:7), (**C**) toluene: acetonitrile: ethyl acetate (9:5:2), and (**D**) ethyl acetate: water: formic acid: acetic acid (100:21:11:11). Each chromatogram is shown under white light, UV254 nm, and UV366 nm before derivatization, and again under white light and UV366 nm after derivatization. Chromatograms (**A**–**C**) were derivatized with anisaldehyde–sulfuric acid reagent, while chromatogram (**D**) was treated with NP/PEG reagent

The system comprising toluene, acetonitrile, and ethyl acetate (9:5:2) produced the most distinct separation pattern (Fig. 2C). Fluorescent zones were clearly visible under UV254 and UV366, with minimal smearing. The bands were distributed across a broad range of Rf values. In contrast, the other two systems (Fig. 2A and B) generated fewer detectable bands, with overlapping zones and poor baseline resolution. The fourth system (ethyl acetate:water:formic acid:acetic acid, 100:21:11:11) revealed polar compounds under UV366; however, visibility declined under white light. The 9:5:2 mixture served as the mobile phase for TLC-bioautography, offering effective resolution across the mid-polarity range. This clarity facilitated the identification of antibacterial zones and their association with specific compounds through visual mapping.

Given their strong antibacterial performance across multiple assays, HKK and MHS were selected for further spatial bioactivity localization. These cultivars showed potent inhibition in disc diffusion and MIC/MBC tests, indicating the presence of active constituents. Moreover, the solvent system optimized via HPTLC (toluene:acetonitrile:ethyl acetate, 9:5:2) was adopted for TLC-bioautography to enable high-resolution mapping of antibacterial bands. This integration of activity-based cultivar selection and chromatographic resolution facilitated effective visualization of bioactive zones.

### TLC-bioautography for bioactivity localization

Bioautography on TLC plates revealed the spatial distribution of antibacterial constituents in 50% ethanolic extracts from *Chrysopogon zizanioides* (MHS) and *C. nemoralis* (HKK). These two cultivars were selected based on their pronounced antibacterial effects, which were confirmed through disc diffusion and MIC/MBC assays.

**Fig. 3:**
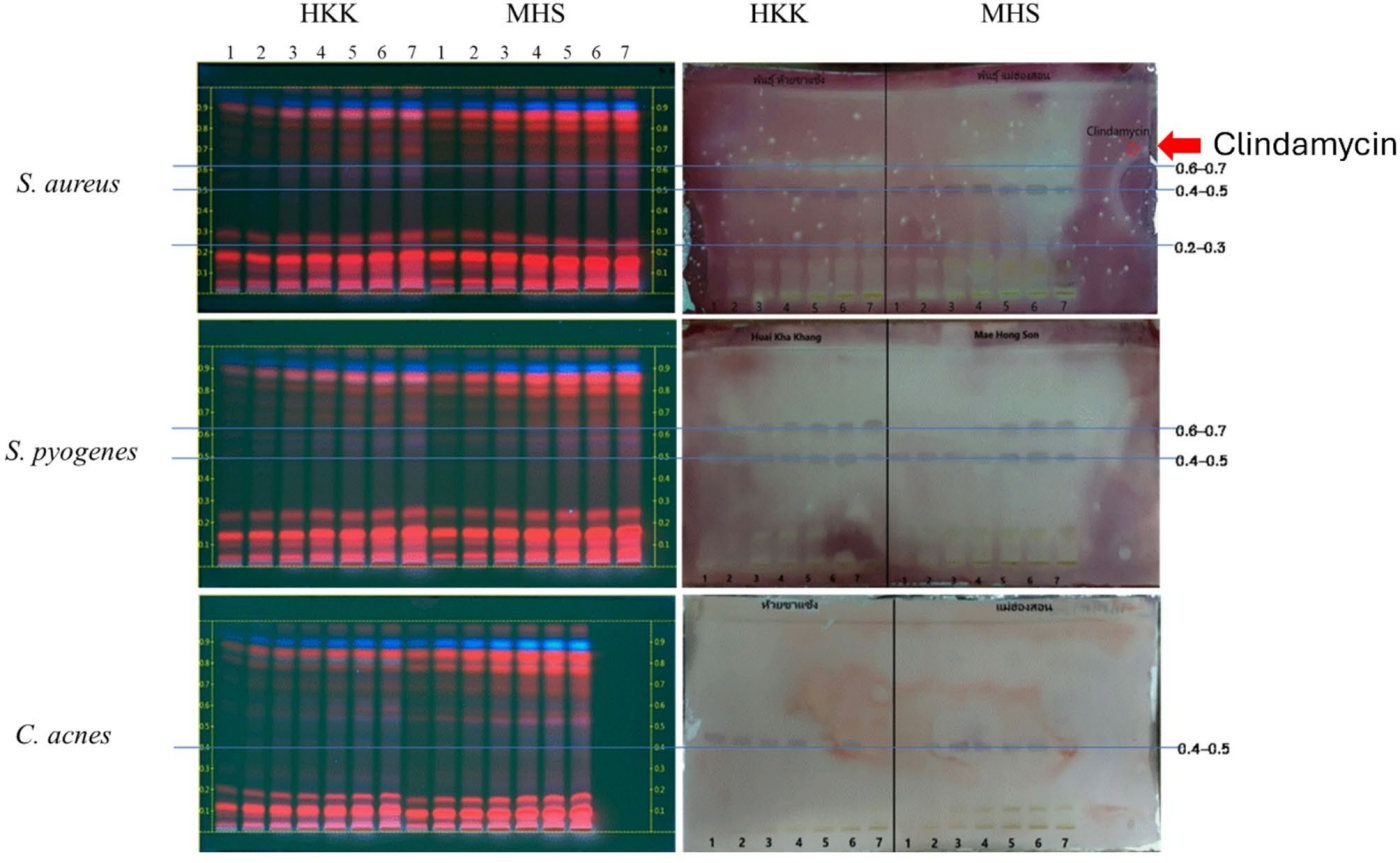
TLC-bioautography of 50% ethanolic vetiver extracts from *Chrysopogon nemoralis* (Huai Kha Khang; HKK) and *Chrysopogon zizanioides* (Mae Hong Son; MHS) against *S. aureus*,* S. pyogenes*, and *C. acnes*. Lanes 1–7 represent extract concentrations from 10 to 70 mg/mL. Chromatograms were developed using toluene: acetonitrile: ethyl acetate (9:5:2) and derivatized with anisaldehyde–sulfuric acid reagent. The left panels show fingerprint patterns under UV366 nm. The right panels display antibacterial inhibition zones under white light following TTC staining. Clear zones at Rf 0.6–0.7, 0.4–0.5, and 0.2–0.3 indicate localized suppression of bacterial metabolism. The positive control, clindamycin (top-right corner), was included for reference. Band color intensity under UV does not directly correlate with antimicrobial activity, as detection was based on TTC-derived colorless zones

The bioautograms showed inhibitory zones at multiple Rf regions, indicating the presence of antibacterial constituents in both plant species. The active compounds that inhibited *S. aureus* were located at three Rf ranges: 0.6–0.7, 0.4–0.5, and 0.2–0.3. For *S. pyogenes*, inhibition zones were detected in the 0.6–0.7 and 0.4–0.5 regions. A single zone at Rf 0.4–0.5 inhibited *C. acnes*. The overlapping inhibition zones across all three bacterial strains in the Rf 0.4–0.7 region suggest that this is the principal range of antibacterial activity (Fig. 3).

Clindamycin, used as a positive control and spotted at the top-right corner of the bioautographic plate, yielded a clear inhibition zone confirming assay validity. While bands observed under UV366 nm were intensely colored, antibacterial activity was determined from colorless zones on the TTC-stained images under white light. Therefore, the bioactivity does not necessarily correlate with chromogenic band intensity, but with TTC-inactive regions indicating growth inhibition.

Following treatment with anisaldehyde–sulfuric acid reagent, the zones at Rf 0.4–0.5 and 0.6–0.7 developed a gray coloration, indicating the possible presence of terpenoid and flavonoid-like compounds. Notably, the consistent appearance of inhibition zones at Rf 0.4–0.5 across all tests highlights their potential importance. Given their moderate polarity, these constituents are considered promising candidates for incorporation into semi-solid topical formulations due to their likely bioavailability.

### Gel formulation optimization and antibacterial evaluation

The optimized gel base (prior to extract incorporation) exhibited high clarity, neutral pH (6.92–7.00), and no sign of phase separation during centrifugation. Base formulations A, B, and C differed in viscosity, texture, and moisturization, with Formulation C showing the highest viscosity. After four weeks of storage under different temperature conditions, Base B demonstrated the best physical stability. Although slight variations in pH and viscosity were observed, the gels maintained stable color, odor, and antibacterial activity throughout the storage period.

These results represent the performance of vetiver gel formulations prepared by incorporating 50% ethanolic extract from *C. nemoralis* (HKK) into the optimized base. Two prototypes (B1 and B2) were created using different extract concentrations. Both appeared gold-brown with a distinct vetiver scent. B1 provided a more noticeable moisturizing sensation, while B2 appeared visually more uniform. In physical characterization, B1 recorded the highest viscosity values, whereas B2 showed a moderate but stable profile. Their pH levels remained between 6.30 and 6.45. Phase separation occurred consistently in B1 across all storage temperatures, especially at 4 °C. In contrast, B2 maintained structural integrity under all tested conditions, suggesting superior formulation stability.

**Fig. 4:**
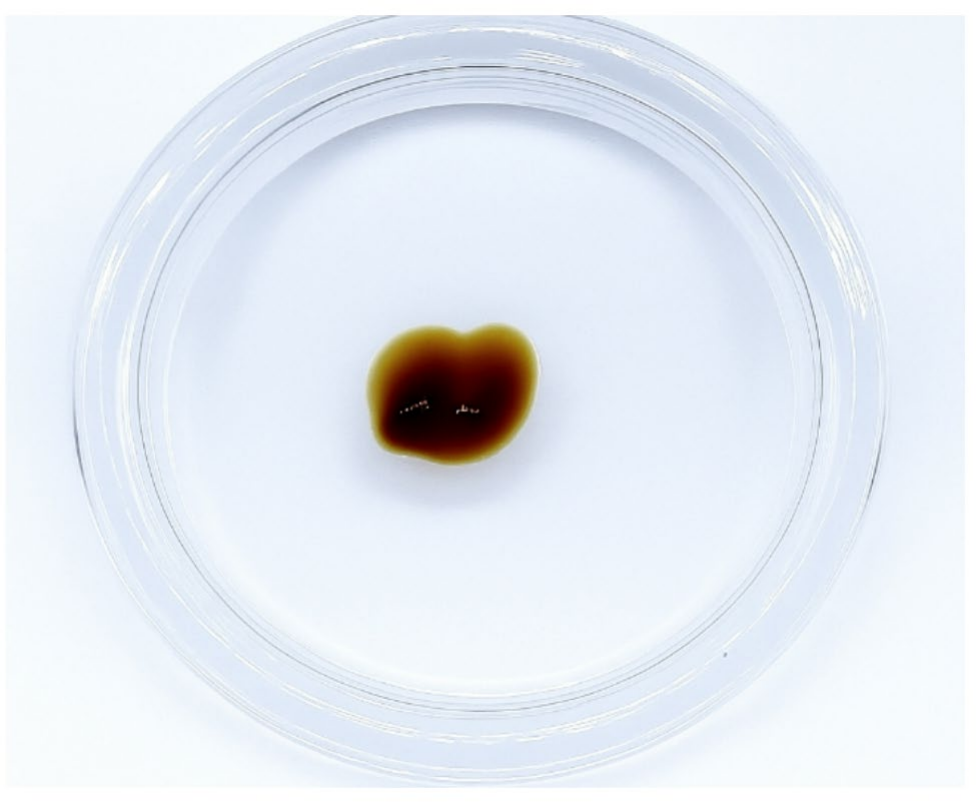
Visual appearance of the optimized antibacterial gel prototype (B2), formulated with 5% ethanolic extract of *Chrysopogon nemoralis* (HKK). The gel exhibited a gold-brown color and uniform consistency after 4 weeks of storage at 25 ± 2 °C

In antibacterial assays, both B1 and B2 exhibited comparable inhibition against *S. aureus* and *S. pyogenes*. However, B2 demonstrated stronger inhibition of *C. acnes*, with consistent activity maintained across all storage conditions (Table 6). The reduced activity of both gels compared to crude extracts may reflect limited diffusion of active compounds from the hydrogel matrix, a known limitation in semi-solid formulations. Additionally, active ingredients may have interacted with or absorbed into the agar, reducing their lateral spread. When benchmarked against standard treatments, inhibition zones of B1 and B2 were markedly smaller than those produced by the 1% clindamycin gel (CG) and the clindamycin disc (CD). Nevertheless, the optimized antibacterial gel prototype (B2), which contained 5% *C. nemoralis* (HKK) extract, showed the most favorable balance of physical stability and antimicrobial efficacy, supporting its selection for further application (Fig. 4).

**Table 6:** Antibacterial activity of two vetiver gel formulations against *S. aureus*,* S. pyogenes*, and *C. acnes*

Sample	Zone of inhibition (mm)		
	*S. aureus*	*S. pyogenes*	*C. acnes*
B1	6.50 ± 0.00^b^	20.33 ± 2.52^b^	9.23 ± 0.30^c^
B2 CG CD	6.50 ± 0.00^b^ 22.00 ± 0.00^a^ 22.50 ± 0.00^a^	20.33 ± 0.58^b^ 35.83 ± 1.04^a^ 35.00 ± 0.02^a^	10.67 ± 2.08^b^ 51.67 ± 1.53^a^ 52.00 ± 0.90^a^

Values are presented as mean ± SD (*n* = 3). Different superscript letters within the same column indicate significant differences (*p* < 0.05) based on Tukey’s HSD test. Comparisons were made within each column

## Discussions

### Novelty and relevance of vetiver leaf-based formulation

This research proposes a novel dermatological application based on underutilized vetiver leaf biomass, which has been historically underutilized compared to the extensively commercialized essential oil from vetiver roots. While vetiver roots have mainly been studied in soil erosion control and production of essential oils, the leaves are commonly left out of high-value applications and are typically routed into low-impact on-farm uses such as mulching (Martinez et al. 2004). By exploring the bioactivity of crude ethanolic extracts obtained from discarded leaves of *C. zizanioides* and *C. nemoralis*, this work expands the medicinal value of a plant commonly used in agriculture. Leaf-derived extracts have demonstrated strong antibacterial action and show promising interactions with cosmeceutical-grade gel base components, which in turn suggest their potential in dermatological formulation research.

The main novelty of this work is the valorization of phytochemical-rich agricultural waste into antimicrobial gel formulations targeting AMR-related skin pathogens. This direction is especially relevant in light of increasing resistance observed in acne-associated bacteria such as *S. aureus*, *S. pyogenes*, and *C. acnes* (George et al. 2022). AMR has been cited in dermatological contexts where repeated antibiotic use may disrupt skin microbiome (World Health Organization 2023; Woo et al. 2023). This observation reflects broader movements advocating plant-based approaches to antimicrobial development worldwide (Dessinioti and Katsambas 2022).

To support this translational approach, phytochemical profiling using HPTLC, combined with bioactivity-guided localization via TLC-bioautography, enabled the identification of active fractions. These were further developed into stable antibacterial gels supporting both value-added utilization and the advancement of AMR-aware topical formulation.

### Extraction efficiency and solvent polarity dynamics

Although extraction yields varied with both solvent type and cultivar, 50% ethanol consistently produced the highest yields across nearly all vetiver leaf samples. This aqueous ethanol mixture functions as a mid-polar solvent capable of dissolving both hydrophilic and lipophilic phytochemicals, particularly low-molecular-weight terpenoids, phenolics, and flavonoids (Mehmood et al. 2022). The SL cultivar generated the highest extract yield (12.40%) when processed with 50% ethanol, whereas MHS produced the lowest output (1.47%) when extracted using 95% ethanol. While solvent polarity was the primary factor influencing yield, some differences between cultivars may arise from leaf morphology and phytochemical content shaped by both genetic and environmental factors (Mehmood et al. 2022). These findings demonstrate that aqueous ethanol is more effective than absolute ethanol in extracting a broader range of bioactive due to enhanced cell wall permeability (Khanal et al. 2022).

In addition to improving extraction yield, the solvent system also shaped antibacterial activity in subsequent assays. Extracts prepared with 50% ethanol provided both greater mass recovery and stronger antimicrobial effects. This suggests that solvent polarity affects not only the quantity of extractable yield but also the composition, which ultimately affects bioactivity. Based on these advantages, 50% ethanol represents a balanced and efficient solvent for generating stable bioactive formulations from plant-based extracts making it ideal for subsequent bioactivity-guided formulation development.

### Antibacterial spectrum and MIC/MBC correlation

Antibacterial activity of vetiver leaf extracts varied according to bacterial strain, solvent type, and vetiver cultivar. The 50% ethanolic extract of HKK (*C. nemoralis*) demonstrated the highest antibacterial activity against both *S. aureus* (17.67 ± 0.29 mm) and *C. acnes* (15.00 ± 1.00 mm) among all tested conditions. The highest *S. pyogenes* growth inhibition was observed with MHS (*C. zizanioides*) extracted using 95% ethanol, which produced a 17.33 ± 2.52 mm zone of inhibition. Stronger antibacterial effects were observed in ethanol-based extracts, which is consistent with their ability to solubilize mid-polar compounds such as flavonoids and terpenoids (Tourabi et al. 2023; Wasihun et al. 2023).

No activity was detected in water-based extracts from any cultivar, reflecting the poor solubility of active molecules in aqueous systems. Multiple studies using herbal plants have established ethanol as an efficient extraction agent for antimicrobial compounds such as phenolics since they have limited water solubility (Hemeg et al. 2020).

MIC and MBC data clarified susceptibility trends across strains. *S. pyogenes* showed the most sensitivity to treatment with MIC values at 7.81 mg/mL while, in contrast, *C. acnes* along with *S. aureus* displayed variability in sensitivity through their MIC range extending to 31.25–125 mg/mL and MBC reaching 500 mg/mL. Previous studies have shown that *S. aureus* is resistant to phytochemical treatments by using both efflux pumps and cell wall barriers as part of adaptive mechanisms (Kalemba and Kunicka 2003).

The consistent performance of 50% ethanolic HKK and MHS extracts supported their selection for TLC-bioautography due to both high activity and consistency across bioassays. The antibacterial response patterns arise from cultivar-specific chemical characteristics and compound interactions that exhibit solvent-dependent behaviors, forming the basis for further separation research and standardization development. These results advance bioactivity-guided phytochemical screening and inform the refinement of sustainable dermatological extraction strategies.

### HPTLC chromatographic fingerprinting

After developing TLC plates using 50% ethanolic extracts, visual distinctions among the five vetiver cultivars were clearly visible. Among the solvent tested, the mobile phase composed of toluene, acetonitrile, and ethyl acetate (9:5:2) yielded the best resolution, producing well-separated bands under UV at both 254 and 366 nm. This mobile phase effectively resolved non-polar and mid-polar phytochemicals, consistent with prior findings on combined aromatic–polar solvent systems in metabolite separation (Gangadharan and Sankararajan 2024).

Comparative fingerprinting revealed both shared and cultivar-specific bands. For instance, high-Rf bands observed in HKK (*C. nemoralis*) overlapped with those in SL (*C. zizanioides*), although intensity differences were especially notable in mid-Rf regions. Chemotaxonomic insights suggest that *Chrysopogon* spp. have a shared biosynthetic background as previously reported in studies comparing chemotypes within the *Chrysopogon* genus, but phytochemical accumulation varies due to cultivar-specific genetics and environmental factors (Gao et al. 2012).

The HPTLC profiles revealed distinguishable chemical patterns among the five vetiver cultivars, enabling consistent comparisons across samples based on constituent distribution. These profiles were also helpful later in selecting solvents for fractionation and tracking the behavior of specific constituents. Overall, HPTLC fingerprinting demonstrates its potential as a quality assessment method for leaf-based natural products within the *Chrysopogon* genus, reinforcing efforts toward standardization and traceability in phytopharmaceutical applications underscoring the role of HPTLC in quality control of standardized herbal dermatological formulations (Vázquez and Tabanca 2025).

### TLC-bioautographic localization of active zones

TLC-bioautography was used to map the antibacterial zones in 50% ethanolic extracts from *C. nemoralis* (HKK) and *C. zizanioides* (MHS). These two cultivars were selected based on their notable antibacterial activity observed in earlier assays. Chromatograms developed with a mobile phase of toluene, acetonitrile, and ethyl acetate (9:5:2) showed inhibition bands at Rf 0.6–0.7 and 0.4–0.5 across all tested pathogens, with an additional zone at Rf 0.2–0.3 found only against *S. aureus*.

After derivatization, bands in the mid-Rf range developed gray coloration, which is commonly associated with sesquiterpenes, flavonoid aglycones, and polyphenols (Wang et al. 2021). There is scientific evidence that compounds from these groups are naturally present in vetiver, particularly in the roots and apical shoots (Arafat et al. 2024; Maurya et al. 2023). This observation suggests that similar phytochemical classes may also be expressed in the leaves. Although the leaf phytochemistry remains underexplored, the alignment of colorimetric responses supports the presence of bioactive constituents from these compound families. While derivatized band colors vary across Rf values due to underlying structural differences, the TTC-detected inhibition zones provide direct evidence of antimicrobial effects. These results indicate that antibacterial activity in vetiver extracts is driven by phytochemical complexity rather than the intensity of individual bands. Such bioactive compounds typically migrate within low to mid-polarity zones during TLC separation. In this study, antibacterial activity was predominantly observed in regions above Rf 0.4, while high-polarity zones exhibited reduced activity, possibly due to lower membrane permeability or weaker interactions with Gram-positive bacterial membranes (Choma and Grzelak 2011; Yichao et al. 2020).

A distinct inhibition zone at Rf 0.2–0.3 was observed only in *S. aureus*. Such selectivity may be influenced by compound abundance or differences in bacterial membrane properties. In addition, both vetiver cultivars showed active zones at similar Rf values, particularly between 0.4 and 0.7. This pattern was also detected in previous reports on root and oil extracts from *Chrysopogon* species (Choma and Grzelak 2011; Sun et al. 2019; Yichao et al. 2020).

There appears to be overlap in antibacterial regions between *C. nemoralis* and *C. zizanioides* leaves, which may reflect underlying chemical similarities. While previous research has focused on root and oil extracts, several of those studies have reported compounds with antimicrobial and antioxidant activity (David et al. 2019; Kim et al. 2005; Oliveira et al. 2022; Pandey and Tiwari 2024). In this study, the antibacterial activity was mapped directly from leaf-based chromatographic profiles. The results confirmed that *C. nemoralis* leaves harbor phytochemicals with demonstrated antimicrobial relevance.

TLC-bioautography was used to identify antibacterial regions on the developed chromatograms. Based on TLC-bioautographic profiles, the HKK extract containing active bands at Rf 0.4–0.7 was chosen for gel formulation. These fractions were aligned based on polarity patterns observed during development which informed the subsequent fractionation and formulation reproducibility steps. Overall, this technique contributes to a more targeted development process for antibacterial agents derived from *C. nemoralis* leaves.

Previous studies have shown that processed *Pluchea indica* leaves can be used to develop nanocomposite materials with antibacterial properties against Gram-positive bacteria through green bioprocessing approaches (Selim et al. 2025). In line with this trend, *C. nemoralis* leaves and other agricultural residues represent promising sources of bioactive compounds for sustainable antibacterial product development. These findings not only confirm antibacterial regions but also provide a rational basis for extracting incorporation into semi-solid systems.

### Physicochemical stability of gel base and vetiver-enriched formulations

Effective formulation of a vetiver-based topical gel depends on integrating antimicrobial activity with physicochemical stability across storage conditions. Among the tested gel bases, formula B maintained consistent texture and stability throughout all settings. The accelerated stability tests confirmed previous findings that ammonium acryloyldimethyltaurate-based systems exhibit thermal resilience and form elastic gel matrices capable of withstanding temperature fluctuations (Cosmetic Ingredient Review Expert Panel 2016) .

Adding crude vetiver extract to the base gel led to the formation of formulations B1 and B2 which exhibited variations in pH and viscosity over time. Both samples showed pH values between 6.30 and 6.45. Products within this range are commonly tolerated by skin and tend not to interfere with its natural barrier or microbial balance. Accelerated breakdown of heat-sensitive compounds in the extract may explain the more pronounced pH shifts observed at 45 °C. Furthermore, the increase in the gel viscosity after four weeks of storage at all temperatures can be attributed to solute–polymer interactions suggesting viscosity increase may be driven more by solute–polymer affinity than merely extract dosage (de Oliveira Pinto et al. 2011).

Of the two formulations, B2 showed greater physical stability under all storage conditions, despite containing a higher quantity of crude extract. Notably, B2 defied expectations, as high phytochemical levels are typically correlated to phase separation and matrix disruption. The stability observed in B2 can be attributed to favorable molecular interactions between bioactive compounds and the polymer matrix, especially polyphenols and flavonoids. These compounds have been reported to participate in hydrogen bonding as well as aromatic stacking interactions, both of which may provide additional cross-linking within the gel matrix. Such secondary interactions might contribute to the formation of more cohesive and durable gel networks, as substantiated by recent structural studies (Feng et al. 2023; Xiao et al. 2023). On the other hand, B1, which had a lower extract concentration, showed early signs of phase separation. This may be due to weak interactions at the molecular level that failed to hold the matrix together. Such evidence highlights that extract levels should be carefully adjusted to maintain both antimicrobial efficacy and physical stability.

Additional evidence suggests that polyphenols serve dual functions in gel systems: as antimicrobial agents and as structural stabilizers. This dual role has been highlighted by Xue et al. (2024), who reported that plant-derived polyphenols contribute to both gel resilience and sustained functionality. To enhance future formulations, the stability of antibacterial activity over time and rheological performance should be evaluated under consumer-relevant conditions. These assessments should be supplemented with bioactive release profiling to ensure clinical efficacy and industrial applicability. Even when crude vetiver leaf extract was used, the gel maintained its structure. This result highlights a practical opportunity to incorporate agricultural residues into dermaceutical systems via bioprocess pathways.

### Antibacterial efficacy of vetiver-based gel formulations

The inclusion of crude vetiver extract in topical gels preserved antibacterial activity against *S. pyogenes*,* S. aureus*, and *C. acnes*, although inhibition zone sizes varied depending on bacterial strain, extract content, and delivery matrix among formulations and bacterial strains. B1 and B2 gels exhibited comparable inhibition against *S. pyogenes*, with both producing zones of 20.33 mm, which aligned with the high susceptibility observed in MIC and disc diffusion assays. The gel base showed no interference with the bactericidal activity; the active components retained their functional integrity after incorporation.

B2 showed stronger antibacterial activity (10.67 ± 2.08 mm) against *C. acnes* compared to B1 (9.23 ± 0.30 mm) with observed data showing a link between dosage level and previous MIC results. Higher extract content may enhance the delivery of active compounds to deeper, anaerobe-rich skin layers by improving their diffusion and retention in skin-mimicking environments. Both formulations showed similar action against *S. aureus*, producing inhibition zones of around 6.5 mm. This matched earlier MIC and MBC findings for the same strain. The thick peptidoglycan wall of *S. aureus*, along with its resistance mechanisms, can limit diffusion of active compounds and reduce their effect when delivered in gel formulations (Xue et al. 2024).

Compared to the 1% clindamycin gel (CG) and the 2 µg clindamycin disc (CD), B1 and B2 formulations produced moderate inhibition zones (Table 6), yet remained less potent than the synthetic control. The advantage of the vetiver-based formulations lies in their botanical origin and broad phytochemical composition, which also reduces the likelihood of resistance development. Although less potent than clindamycin, the vetiver-based gels offer a plant-derived alternative with favorable skin compatibility and a lower risk of promoting AMR. Their polypharmacological nature and mild formulation matrix further support long-term use without compromising skin health (Iraji et al. 2021).

Both gels remained effective after four weeks across all storage conditions. A comparable outcome had been seen previously in studies involving vetiver-based lotions (Kurrimboccus et al. 2022). In this study, antibacterial activity was evident against skin-associated bacteria, including strains linked to acne. Further work may focus on how the formulations interact with the skin and function under physiological conditions. These investigations could clarify whether the gels are suitable for practical therapeutic use.

### Strategic positioning and future application

Among the five *Chrysopogon* cultivars examined, *C. nemoralis* (HKK) showed the most potent antibacterial activity. This study provides one of the first integrative investigations combining TLC-bioautography with formulation science using *C. nemoralis* leaf extract, enabling a direct link between phytochemical profiles and bioactivity in a dermatological context.

Instead of relying on volatile oils, this study focuses on mid-polar, non-volatile compounds such as sesquiterpenes and flavonoid aglycones which are better suited for gel formulations. Using extractable content from discarded leaves, it turns unused plant material into functional ingredients for skin-related applications. This process supports the reuse of agricultural byproducts and fits into ongoing efforts to develop natural alternatives in skincare products that avoid synthetic antimicrobials.

Chromatographic fingerprinting, TLC-bioautography, and formulation science were integrated to ensure that biological efficacy is preserved while maintaining chemical traceability and scalability. These elements position the formulation as a viable AMR-conscious cosmeceutical candidate for future clinical translation and address growing concerns over the use of synthetic preservatives in topical products (Salam et al. 2023). Given its stability in accelerated storage conditions, the developed formulation may proceed to further testing, including skin permeation, irritation, and dermatokinetic evaluation in preclinical models.

Among the tested gels, B2 showed stable antibacterial results and kept its texture steady during storage. It was effective against *C. acnes*, *S. pyogenes* and also reduced *S. aureus* to a lesser degree. The gel maintained a skin-compatible pH and stayed physically stable, which suggests possible use in products like acne treatments, post-shave applications, and botanical wound care. In the next steps, identification and characterization of the main components in the Rf bands and improving extraction methods based on accepted safety requirements will be critical for future therapeutic applications. This type of formulation may help individuals who need alternatives to repeated antibiotic use.

TLC-bioautography helps locate antibacterial compounds on the plate and provides an early indication of their potency before purification. In one study, lower Rf bands showed varying antibacterial intensity at different concentrations (Jesionek et al. 2019). Another group applied TLC-bioautography to detect antifungal activity in *Lupinus* plant extracts (Cely Veloza et al., 2023). To verify compound identity and fulfill regulatory criteria, further analysis using LC-MS/MS and NMR will be needed (Gaudêncio et al. 2023; European Medicines Agency, 2025).

This research builds a phytochemical foundation for innovation in topical therapies targeting skin conditions linked to AMR. The growing interest in botanical antimicrobials has emphasized the value of agricultural residues, particularly underused leaf biomass. Scientists are now turning their attention to botanical sources of therapeutic compounds which include understudied plant materials found in various agricultural waste products (Bala et al. 2023; Garg 2023; Machado et al. 2024). Use of such valorization techniques in bioprocessing systems provides researchers and formulators with opportunities to enhance sustainability in product development. This incorporation marks the convergence of phytochemical utilization and sustainable skincare innovation, highlighting the untapped potential of agricultural residues like *C. nemoralis* leaves.

## Conclusion

This study demonstrates a bioactivity-guided approach for valorizing underutilized *Chrysopogon nemoralis* leaf biomass into a stable antibacterial gel. Among the five tested cultivars, the 50% ethanolic extract of the Huai Kha Khaeng (HKK) showed the strongest antibacterial activity, particularly against *Cutibacterium acnes* and *Streptococcus pyogenes*, while exhibiting moderate inhibition of *Staphylococcus aureus*. TLC-bioautography enabled the localization of bioactive zones associated with mid-polar constituents, which guided the selection of extract fractions for gel formulation.

A 5% w/w topical gel was developed and remained stable while retaining antimicrobial efficacy under varied storage conditions. This work outlines a stepwise framework involving sustainable extraction, targeted screening, and functional product development, highlighting its translational potential for natural skincare innovation.

Future research should focus on the characterization of bioactive compounds, extract optimization, and compliance with regulatory standards. Overall, this study supports a scalable and sustainable strategy for converting agricultural residues such as vetiver leaves into plant-based antimicrobials for dermatological care where antimicrobial resistance is a growing concern.

## Data Availability

The datasets used and/or analyzed during the current study are available from the corresponding author on reasonable request.
